# A new biomarker candidate for spinal muscular atrophy: Identification of a peripheral blood cell population capable of monitoring the level of survival motor neuron protein

**DOI:** 10.1371/journal.pone.0201764

**Published:** 2018-08-13

**Authors:** Noriko Otsuki, Reiko Arakawa, Kaori Kaneko, Ryoko Aoki, Masayuki Arakawa, Kayoko Saito

**Affiliations:** 1 Institute of Medical Genetics, Tokyo Women’s Medical University, 10-22 Kawadacho, Shinjuku-ku, Tokyo 162-0054, Japan; 2 Affiliated Field of Medical Genetics, Division of Biomedical Engineering and Science, Graduate Course of Medicine, Graduate School of Tokyo Women’s Medical University, 10-22 Kawadacho, Shinjuku-ku, Tokyo 162-0054, Japan; 3 Laboratory of Virology, Institute of Microbial Chemistry, 5-14-23 Kamiosaki, Shinagawa-ku Tokyo 141-0021, Japan; University of Minnesota Medical Center, UNITED STATES

## Abstract

Spinal muscular atrophy (SMA) is a severe genetic neuromuscular disorder caused by insufficiency of functional survival motor neuron (SMN) protein. Several clinical trials have been conducted with the aim of upregulating the expression of the SMN protein in SMA patients. In order to evaluate the efficiency of these SMN-targeted approaches, it has become necessary to verify SMN protein levels in the cells of SMA patients. Accordingly, we have developed a method allowing the evaluation of the functional SMN protein with < 1.5 mL of peripheral blood using imaging flow cytometry. The expression of SMN protein in CD3^+^, CD19^+^, and CD33^++^ cells obtained from SMA patients, was significantly reduced compared with that in cells from control subjects. In spot analysis of CD33^++^ cells, the intensities of SMN spots were significantly reduced in SMA subjects, when compared with that in controls. Therefore, SMN spots implied the presence of functional SMN protein in the cell nucleus. To our knowledge, our results are the first to demonstrate the presence of functional SMN protein in freshly isolated peripheral blood cells. We anticipate that SMN spot analysis will become the primary endpoint assay for the evaluation and monitoring of therapeutic intervention, with SMN serving as a reliable biomarker of therapeutic efficacy in SMA patients.

## Introduction

Spinal muscular atrophy (SMA) is an autosomal recessive neuromuscular disorder characterized by the destruction of motor neurons in the anterior horn of the spinal cord. It is caused by deletion or mutation of *survival motor neuron 1* (*SMN1*) encoding the SMN protein [1]. SMA presents as a continuous spectrum of symptoms classified into four subtypes on the basis of age at onset and achievement of motor milestones [2]. Type I SMA, known as Werdnig–Hoffmann disease, is the most severe form of the disease. Among individuals with Type I SMA, disease onset occurs within six months after birth. Sufferers are unable to sit without support. Most infants with this disease survive for less than 24 months without ventilator support. Type II SMA, otherwise known as Dubowitz disease, is an intermediate form. The disease onset in individuals with Type II SMA occurs between six and 18 months. Infants with this disease are able to maintain a sitting position, but are unable to stand or walk without support. However, their lifespans are expected to extend into adulthood. Type III SMA, otherwise known as Kugelberg–Welander disease, is a milder form. The disease onset in individuals with Type III SMA occurs after 18 months of age. Infants with Type III SMA are able to walk before the onset of disease, but gradually develop symptoms. The average lifespan of individuals presenting with Kugelberg–Welander disease does not differ from that of the general population. The onset of Type IV SMA, which is often observed as a sporadic disease, is after 20 years of age. The symptoms of Type IV disease gradually progress over several decades.

Humans have two forms of SMN protein-coding genes located on chromosome 5q13, identified as *SMN1* and *SMN2*. *SMN1* and *SMN2* are almost identical. However, 70%–95% less of the full-length SMN protein is produced by *SMN2* than by *SMN1*. The reason for *SMN2* being unable to produce sufficient amount of full-length SMN protein is a C–T replacement inside exon 7 that is not present in *SMN1*. This replacement does not affect the amino acid sequence, but does result in alternative splicing, and the skipping of exon 7 [3, 4]. This results in the production of a truncated mRNA lacking exon 7 (*SMN*Δ*7*). *SMN*Δ*7* encodes SMNΔ7 protein, which inhibits the general actions of the SMN protein, including oligomerization, complex formation, and stabilization [5, 6]. Most SMA patients harbor genetic mutations in *SMN1* and have more than two copies of *SMN2*. An increased copy number of *SMN2* is reported to be strongly associated with an SMA phenotype, and the relationship between the *SMN2* gene copy number and the amount of SMN protein in peripheral blood nuclear cells (PBCs) has been analyzed using a sandwich enzyme-linked immunosorbent assay (ELISA) [7] and electrochemiluminescence immunoassay (ECLIA) [8].

The SMN protein is a ubiquitously expressed intracellular protein implicated in multiple cellular functions, including biogenesis of small nuclear ribonucleoprotein (snRNP) [9, 10], pre-mRNA splicing [11], apoptosis [12], autophagy [13], and axonal formation [14, 15]. However, the cause for the selective degeneration of motor neurons due to the decrease in the amount of full-length SMN protein remains unclear.

Currently, Nusinersen, marketed as “Spinraza^®^” became the first approved drug for SMA in 2016. As potential approaches for therapeutic strategies against SMA, several investigators have attempted to systemically upregulate the levels of the SMN protein, particularly in the anterior horn cells of the spinal cord [16] However, the spinal cord, which is the target organ in SMA therapy, cannot be sampled for the evaluation of therapeutic efficacy. Therefore, it is essential to establish reliable verification methodology that includes the performance of minimally invasive treatments and accurately reflects the systemic level of the SMN protein. Prior studies have evaluated the SMN protein levels in various cells and tissues [7, 8, 17–22].

Herein, we report the establishment of a semi-quantitative analysis of the SMN protein in peripheral blood nuclear cells (PBCs) using imaging flow cytometry. We successfully identified putative target cells in PBCs, which contained functional SMN protein, and thus had the potential of acting as a biological marker allowing assessment of SMA therapeutic interventions [23, 24]. Our findings also shed light on the relationship between the pathological condition of SMA, and the systemic levels of SMN protein in patients during long-term clinical trials.

## Materials and methods

### Study population

All subjects were recruited at Tokyo Women’s Medical University (TWMU: Shinjuku, Tokyo, Japan) under Tokyo Women’s Medical University Institutional Review Board Approved protocols No.2466 and No.3323. All procedures were conducted according to the principles described in the Declaration of Helsinki. Written informed consent was obtained from the subjects and/or their legal guardians as per institutional guidelines. Twenty-five individuals with SMA, ranging in age from 0.2 to 60 years, were enrolled at the Institute of Medical Genetics, TWMU. Prior to diagnosis, the copy number of exon 7 and exon 8 on both of *SMN1* and *SMN2* of all SMA subjects were analyzed. In parallel, physical abilities of the SMA subjects were assessed using the Hammersmith Functional Motor Scale (HFMS) and the Children’s Hospital of Philadelphia Infant Test of Neuromuscular Disorders (CHOP INTEND). Individuals with SMA type I–III were classified into the clinical subtypes for this study, based on individual motor milestone changes, and were assessed as we previously reported [25] (Table 1, *n.d.: not determined*).

**Table 1:** Donor information; ID, Age, Sex, SMA typing, the score of Hammersmith Functional Motor Scale (HFMS), and copy number of *SMN2*.

Subject ID	Age	Sex	SMA subtype	HFMS	*SMN2* Exon7
S1	0y2m	F	Ia	0	2
S2	1y2m	M	Ib	2	2
S3	2y2m	M	Ib	1	2
S4	2y10m	M	Ib	0	2
S5	3y9m	F	IIa	18	3
S6	3y10m	F	IIa	2	2
S7	5y	M	IIa	6	3
S8	7y	M	IIa	18	3
S9	7y	F	IIa	20	3
S10	21y	M	IIa	3	2
S11	41y	M	IIa	*n.d*.	2
S12	2y3m	M	IIb	4	3
S13	3y	F	IIb	4	2
S14	3y2m	M	IIb	12	3
S15	3y5m	M	IIb	24	3
S16	4y	F	IIb	16	3
S17	4y7m	M	IIb	4	3
S18	6y	M	IIb	24	3
S19	8y	F	IIb	*n.d*.	3
S20	13y	F	IIb	*n.d*.	3
S21	14y	F	IIb	*n.d*.	3
S22	7y	F	IIIa	37	3
S23	8y	F	IIIb	36	3
S24	51y	M	IIIb	40	3
S25	60y	F	IIIb	40	4

Infant control subjects were selected from among patients without genetic disorders, and with no oral administration of drugs. Adult control subjects were randomly selected at the Institute of Medical Genetics from volunteers without apparent diseases.

### Cells

In all cases, peripheral blood was collected in the presence of sodium heparin. Blood samples were maintained at room temperature (15°C–26°C) and shaded from light until processed. The day following blood collection, 1.5 mL of blood sample was dispensed into a 15-mL conical tube. As an Fc-receptor blocking reagent for human blood, 30 *μ*L of normal human Ig solution (Clear Back, MTG-001, MBL, Nagoya, Japan) was added to the dispensed blood, and incubated for 15 min at room temperature. Then, fluorochrome-conjugated mAbs against CD33, CD3, and CD19 were added in order to classify the cell populations. After incubation for 30 min at room temperature, the blood samples were treated with 10 mL of pre-warmed Lyse/Fix Buffer (BD Biosciences, San Jose, CA) and incubated at 37°C in a water bath for 10 min. Cells were centrifuged at 900 × *g* and the supernatant was aspirated. Cells were washed with 15 mL of PBS (−) (Ca^2+^ and Mg^2+^ free phosphate buffered saline) one time, and the supernatant was removed. Fixed and washed cells were permeabilized with 2 mL of Perm/Wash Buffer I (BD Biosciences) for 30 min at room temperature. After permeabilization, the cells were washed with Perm/Wash Buffer I and counted using a cell counter (Sceptor^®^, Merck). Cell concentrations were adjusted, and 1 × 10^6^ cells in 50 *μ*L of Perm/Wash Buffer I were dispensed into each of two 1.5-mL microtubes. The cells were stained with mAbs against human SMN and/or human Smith core proteins, and incubated for 45 min at room temperature. Stained cells were washed 3 times (500 × *g*) with 500 *μ*L of Perm/Wash Buffer I and resuspended in 50 *μ*L of Hoechst 33342 (0.25 *μ*g/mL, Molecular probe, Eugene, OR) for 15 min. After incubation, 500 *μ*L of PBS (−) was added to the cells, followed by centrifugation. Washed cells were resuspended in 50 *μ*L of PBS (−) and analyzed using imaging flow cytometry.

### Antibodies

Alexa Fluor 488 (AF488)-conjugated 2B1 (anti-SMN mAb, murine IgG1, *κ*), was purchased from Novus Biologicals (Littleton, CO). Biotinylated Y12 (anti-Smith core proteins mAb, murine IgG3, *κ*) was purchased from Thermo Scientific (Fremont, CA). R-Phycoerythrin (PE)-conjugated mouse anti-human CD19 mAb (HIB19, IgG1, *κ*), R-Phycoerythrin-Cyamin 5 (PE-Cy5)-conjugated mouse anti-human CD33 mAb (WM53, IgG1, *κ*), Brilliant Violet 610 (BV610)-conjugated mouse anti-human CD3 (UCHT1, IgG1, *κ*), BV510-conjugated mouse anti-human CD45 mAb (HI30, mouse IgG1, *κ*), mouse anti-human CD15 mAb (W6D3, IgG1, *κ*), a cocktail of anti-human lineage marker mAbs (for CD3, CD14, CD16, CD19, CD20, CD56), BV785-conjugated mouse anti-HLA-DR mAb (L243, IgG2a, *κ*), mouse anti-human CD14 mAb (M5F2, IgG2a, *κ*), PE-conjugated mouse anti-human CD66ace mAb (ASL-32, IgG2b, *κ*), mouse anti-human CD66b mAb (G10F5, IgM, *κ*), mouse anti-human CD34 mAb (581, IgG1, *κ*), BV650-conjugated mouse anti-human CD11c mAb (3.9, IgG1, *κ*), and isotype controls, including fluorochrome-conjugated mouse IgG1, *κ* (MOPC21), IgG2a, *κ* (MOPC-173), IgG2b, *κ* (MPC-11), and IgM, *κ* (MM30), were all purchased from Biolegend (San Diego, CA). PE-conjugated streptavidin (BD Biosciences) was used as a secondary antibody.

### Imaging flow cytometry

Stained cells (2 × 10^7^ cells/mL) in 50 *μ*L of PBS (−) were acquired using imaging flow cytometry (ImageStreamX Mark II, Merck, Darmstadt, Germany) with two cameras, a 60 × magnification objective lens (1 pixel = 0.3 *μ*m × 0.3 *μ*m), and 12 detection channels controlled by INSPIRE software (Merck). The raw image files were analyzed using IDEAS software (Merck). Samples with a bright field (BF) area lower than 60 pixels (5.4 *μ*m^2^) were gated out to eliminate debris. A minimum of 5,000 events, primarily involving the minor cell population (CD33^++^ or CD19^+^), were collected per sample. The raw image files (.rif) were converted to data analysis files (.daf) using the compensation matrix file (.ctm). For the SMN spot analysis, we chose the nomenclature of “SMN spot” (see Supporting S1 and S2 Figs online). The definition can be summarized as follows: 1) The SMN spot was detected within the nuclear area. 2) The intensity of the maximum bright area of the SMN spot was more than 5.5 times that of background. 3) The areas of SMN spots were defined as ranging between 0.34 and 8.55 *μ*m^2^. 4) The shapes of SMN spots were defined based on the minor axis to the major axis ratio being 0.4 to 1.0. The details of the analysis protocol, the analytical procedure, and the algorithm design used for detecting SMN spots are described online in Supporting S1 and S2 Figs.

### Verification of fluorescence intensity

Polystyrene microparticles (BD^™^ CompBeads; BD Biosciences), which bind specifically to mouse immunoglobulin light chain (*κ*), were used according to the manufacturer’s recommendation. Briefly, 50 *μ*L of the microparticle suspension was transferred into 1.5-mL microtubes (n = 22). The anti-SMN mAb AF488-2B1 was diluted with 2% fetal bovine serum and 0.09% NaN_3_ containing PBS (Stain buffer; BD Biosciences). Pre-diluted (2-fold) mAbs (20 *μ*L) were added to each tube. The final mAb concentrations in each microparticle suspension were 178.6, 89.3, 44.6, 22.3, 11.2, and 0 ng/mL, respectively. Each suspension was conducted in triplicate. All microtubes were tapped, mixed well, and incubated for 30 min at room temperature in the dark. After incubation, 1.5 mL of the staining buffer was added to each of the tubes. Stained microparticles were pelleted by centrifugation at 200 × *g* for 10 min, and resuspended in 50 *μ*L of PBS(−). The microparticles were acquired under the same conditions as those used for the analysis of PBCs by imaging flow cytometry. A minimum of 1,300 events in the strictly focused population of microparticles were collected per sample.

### *SMN1*, *SMN2*, and other related genes copy number analysis

Gene expression phenotypes were determined using SALSA multiplex ligation-dependent probe amplification (MLPA) kit P201-A2 (MRC-Holland, Amsterdam, Netherlands) [26]. This kit contains a mixture of various nucleotide probes specific for SMN-related genes, including exon 7 and exon 8 of *SMN1* (NM_000344); exon 7 and exon 8 of *SMN2* (NM_017411); exons 1, 4, 6, and 8 of *SMN1* and *SMN2*; exon 5 and exon 13 of *NAIP*; *GTF2H2*; *RAD17*; *SERF1B*; and other reference probes. After multiplex ligation-dependent probe amplification, the DNA fragments were analyzed using an Applied Biosystems 3130 Genetic Analyzer (Thermo Fisher Scientific, Waltham, MA) with GeneMapper software ver. 4.1 (Thermo Fisher Scientific).

### Statistical analysis

Unpaired equal variance of the data was assessed by applying a two-sided Student’s t test (*p < 0.05, **p < 0.01, and ***p < 0.001). The differences in the slopes of the regression curves were assessed by analysis of covariance test (ANCOVA, *p* > 0.05). For the comparison within the SMA subjects, unpaired analytical data was assessed by the Brunner Munzel test using the statistical software “HAD” (HAD on16), which is available online (http://jmic-weblab.org/ojs/index.php/jmic/article/view/6/5). Multigroup test was assessed by Spearman’s rank correlation test, Kruskal–Wallis test, and Mann Whitney U test with subsequent Holm’s procedure (*p < 0.05, **p < 0.01, and ***p < 0.001). Trend test of continuous variables was assessed by Jonckheere–Terpstra test (*p < 0.05) using the statistical software “EZR” (Easy R, version 1.36), which is available online (http://www.jichi.ac.jp/saitama-sct/SaitamaHP.files/statmed.html).

## Results

### Determination of the cell populations to be analyzed

**Fig 1:**
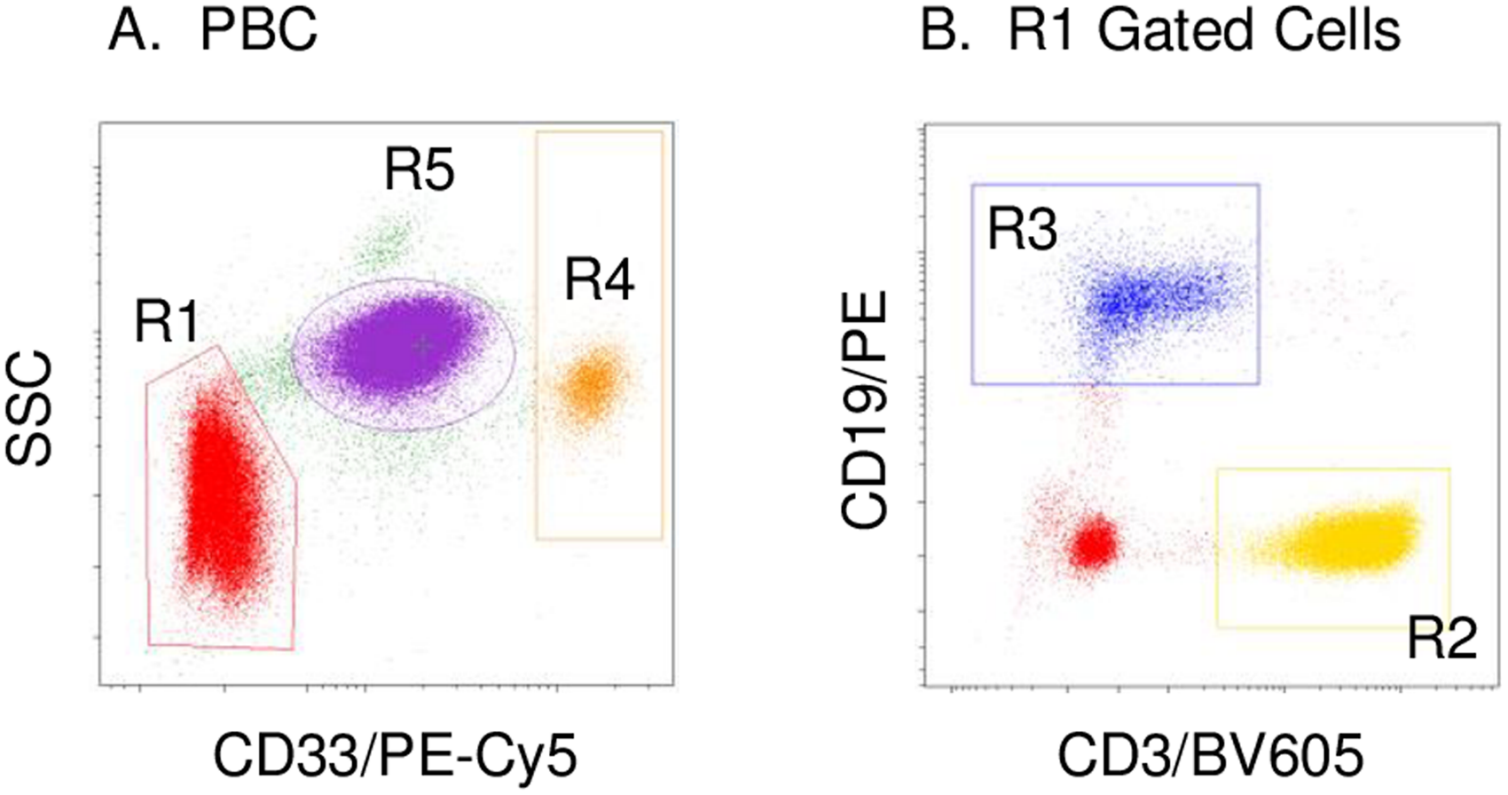
Determination of peripheral blood nuclear cell (PBC) populations. (A) PBCs were analyzed using an anti-CD33 mAb (x-axis) and side scatter (SSC: y-axis). (B) R1-gated cells were analyzed using an anti-CD3 mAb (x-axis) and an anti-CD19 mAb (y-axis). The phenotypes of R2, R3, R4, and R5-gated cells are detailed in Supporting S1 Table online.

Previously, we reported data suggesting a difference in the level of SMN protein expression between SMA subjects and control subjects in cultured fibroblasts [27] and Epstein–Barr virus transformed B cells [28]. In accordance with our prior results, we attempted to compare SMN protein expression in freshly isolated PBCs between SMA subjects and control subjects using imaging flow cytometry. Since PBCs comprise heterogeneous cell populations, we performed analyses to identify the appropriate cell population based on the expression of surface molecules. As shown in Fig 1*A*, PBCs were roughly classified into three groups by specific intensities of sidescatter (SSC) and CD33 (R1, R4, R5). In addition, CD3^+^ cells (R2) and CD19^+^ cells (R3) were included among the R1-gated cells (Fig 1*B*). The expression patterns of surface molecules on classified cells are detailed online in Supporting S1 Table. Thus, we attempted to analyze the expressions of SMN protein in PBCs by dividing the cells into four populations, comprised mainly of T cells (R2), B cells (R3), monocytes (R4), and neutrophils (R5).

### Verification of fluorescence intensity using imaging flow cytometry

**Fig 2:**
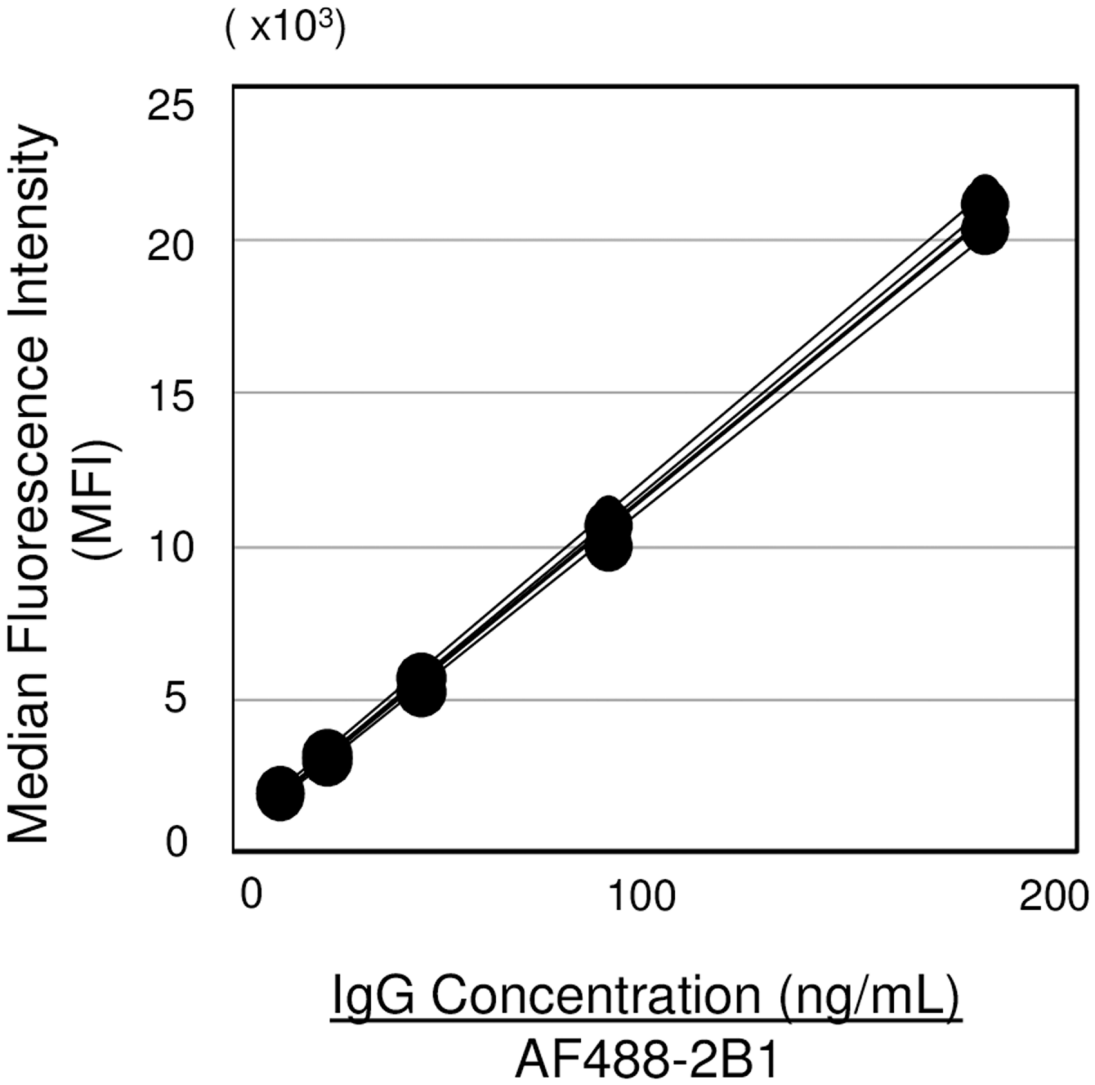
Verification of the correlative evaluation between antibody concentration and fluorescence intensity using imaging flow cytometry. Diluted AF488-2B1 binds specifically with polystyrene beads. The overlaid plots are obtained from five interday analyses (see Supporting S2 Table, online). There were no significant differences among the five scatter plots, as tested by analysis of covariance (ANCOVA) (*p* = 0.9208).

Flow cytometry is not suitable for the absolute quantitative determination of proteins. It is difficult to compare fluorescence intensities among different individuals or at different time points during an SMA patient’s clinical course of disease. To overcome this, we attempted to verify the correlation between median fluorescence intensity (MFI) and antibody concentration in the staining of microparticles with AF488-conjugated anti-human SMN mAb (2B1), both intra-day (data not shown) and inter-day (see Supporting S2 Table, online), using imaging flow cytometry. The coefficient values (CV) of the MFI at each IgG concentration among inter-day data were less than 5% (2.2%–4.7%). These results indicated that sufficient data were obtained as a validation test for the precision of quantitative analysis. The slope values of the regression curves obtained from five independent experiments were similar (CV = 2.0%). Further evaluation by analysis of covariance (ANCOVA) indicated that the shapes of the regression curves did not differ significantly among the five independent experiments (Fig 2, *p* = 0.9208). These data support the possibility of being applicable to semiquantitative analysis of the frequency of SMN expression by maintaining accuracy control of imaging flow cytometry.

### The levels of SMN protein expression

**Fig 3:**
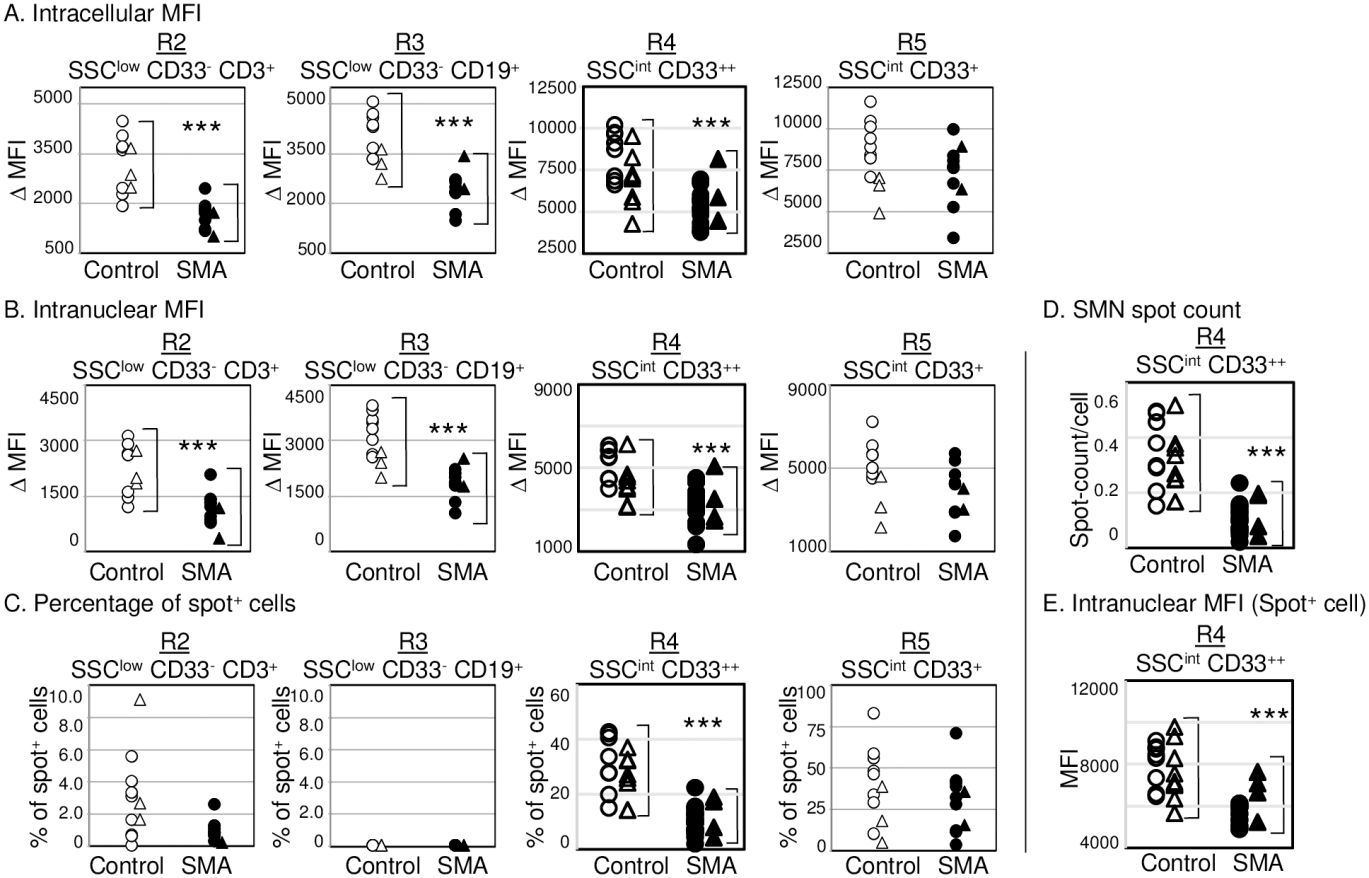
Relative comparison of survival motor neuron (SMN) protein expression between the control and spinal muscular atrophy (SMA) subjects. Peripheral blood nuclear cells (PBCs) classified according to types (R2, R3, R4, R5)were analyzed for the levels of SMN protein expressions in (A) intracellular and (B) intranuclear cells. (C–E) SMN protein spot analysis. (C) The percentage of SMN spot^+^ cells is equal to “the percentage of spot^+^ cells in SMN-stained cells” minus “the percentage of spot^+^ cells in isotype control-stained cells”. The percentage of spot^+^ cells of isotype control-stained cells was < 1.0% (R4 and R5). (D) The mean of SMN spot count per single cell and (E) intranuclear MFI of spot^+^ cells in the CD33^++^ cell (R4). Cells were obtained from 14 control subjects (open symbols) and 25 SMA subjects (closed symbols). Circles indicate infant subjects and triangles indicate adult subjects. ***p < 0.001. The algorithm for SMN spot analysis is detailed in Supporting S2 Fig online.

To examine the levels of SMN expression in the four classified cell populations, we analyzed multicolor stained cells. SMN was expressed in all populations of PBCs, including the R2-, R3-, R4-, and R5-gated cells, in both the control and the SMA groups (Fig 3*A*). When comparing these groups, intracellular SMN expression levels in the R2, R3, and R4 populations from the SMA subjects were found to be significantly reduced compared with those from the control subjects (Fig 3*A*: *p* < 0.001). In the R5 population, the levels of SMN expression varied among individuals in both the control subjects and the SMA subjects. In addition, analysis restricted to the nuclear region, based on Hoechst 33342 fluorescence, provided results similar to those shown in Fig 3*A* for all lymphocyte populations of cells (Fig 3*B*: R2, R3, R4) with statistically significant differences among the different cell populations (*p* < 0.001). There were no significant differences in the data between adults and infants, for either control or SMA subjects. These results indicate that the levels of SMN protein expression in PBCs can be compared using imaging flow cytometry. This approach revealed that the levels of SMN protein expression in PBCs from the SMA subjects was significantly reduced compared with those from the control subjects in the populations of CD3^+^ cells (R2), CD19^+^ cells (R3), and CD33^++^ cells (R4).

### SMN spot analysis

The SMN complex reportedly concentrates in multiple nuclear puncta termed Gemini of Cajal bodies [29]. In our current study, SMN was observed as diffuse, and dotted staining images in both the cytoplasm and the nuclei of PBCs by imaging flow cytometry analysis. When viewing the image files, SMN spots were clearly recognized in CD33^++^ cells (R4). For objective assessment, an algorithm defining the detection of the SMN spots was applied, as detailed in the online Supporting S2 Fig. In the process of establishing the definition, we validated the reproducibility of the SMN spot-detection method using CD33^++^ cells. The results are shown in the online Supporting S3 Table from five independent experiments using cells from three adult control subjects. The relative standard deviations (RSDs) of the median fluorescence intensity of the cell and nuclei (ΔMFI) were 18.8%–40.2%. In comparison, the RSDs of the spot analysis were 1.6%–13.5%. Of note, the RSDs of the percentages of SMN-spot^+^ cells were less than 10.0% (6.6%–10.0%). These data suggest that the SMN spot analysis using imaging flow cytometry was a highly reproducible method for the analysis of SMN protein in sorted PBC samples. As shown in Fig 3*C*, a percentage of SMN-spot^+^ cells were detected in the populations of CD33^++^ cells (R4), and CD33^+^ cells (R5). However, the percentages of SMN-spot^+^ cells significantly reduced compared with those from control subjects (*p* < 0.001) only in the population of CD33^++^ cells obtained from the SMA subjects. As shown in Fig 3*D* and 3*E*, the population of CD33^++^ cells from SMA subjects showed significantly reduced means of SMN spot count per single cell (Fig 3*D*) and intranuclear MFI for the SMN-spot^+^ cells (Fig 3*E*), compared with those of control subjects (*p* < 0.001). There were no significant differences in the data between adults and infants in either the control or the SMA subjects. These data suggest that stable and detectable SMN spots were restricted to SSC^int^ CD33^++^ cells (R4). In particular, the patterns of SMN protein expression based on the percentage of SMN-spot^+^ cells, the mean SMN spot count, and the intranuclear MFI of SMN-spot^+^ cells in CD33^++^ cells from SMA subjects were significantly reduced, compared with those of cells from control subjects (Fig 3*C*, 3*D* and 3*E*).

### Analysis of SMN spot intensity

**Fig 4:**
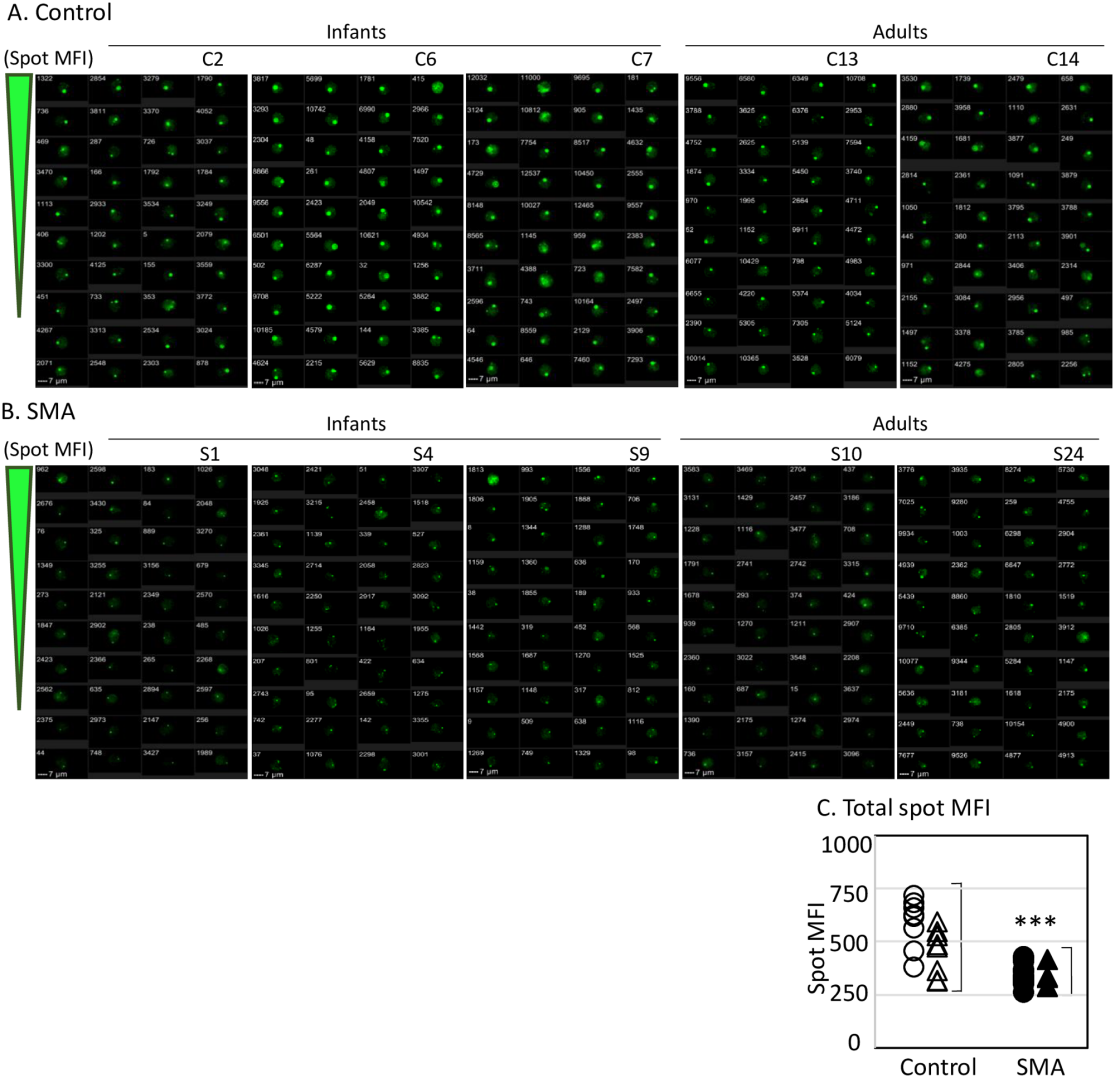
Analysis of survival motor neuron (SMN)-stained images of the CD33^++^ cell population. In total, 40 cells were extracted from the SMN spot with the highest intensity. The numbers at the upper left represent individual numbers, depending on the detection order. (A) Control subjects and (B) Spinal muscular atrophy (SMA) subjects. Three representative images of infants are shown (*left*). Two representative images of adults are shown (*right*). Image panels represent typical examples. (C) Relative comparison of MFI limited to the area of the SMN spots between the control and SMA subjects. Cells were obtained from 14 control subjects (*open symbols*) and 25 SMA subjects (*closed symbols*). Circles indicate infant subjects (control: *eight*, SMA: *tewnty-one*) and triangles indicate adult subjects (control: *six*, SMA: *four*). ***p < 0.001. The numerical data of all subjects are provided in Supporting S4 Table online.

Not only the SMN spot count but also the fluorescence intensities of SMN spots within the cells obtained from SMA subjects were reduced compared with those obtained from control subjects. In Fig 4, the panels of representative images show results from both the control (Fig 4*A*) and the SMA (Fig 4*B*) subjects. Although no difference in the brightness of the SMN spots was observed between the infants (Fig 4*A* and 4*B*, *left*) and adults (Fig 4*A* and 4*B*, *right*) for either control or SMA subjects, the brightness of the SMN spots for SMA subjects (Fig 4*B*) was reduced compared with control subjects (Fig 4*A*). For numerical evaluation, determination of MFI limited to the area of the SMN spots, termed “SMN spot MFI”, was calculated by applying an SMN spot mask (see online Supporting S4 Table, “SMN Spot MFI”). These data were plotted in the graph shown in Fig 4*C*. SMN spot MFI in CD33^++^ cells obtained from SMA subjects was significantly reduced compared with CD33^++^ cells from control subjects. These data indicate that the SMN localization in CD33^++^ cells from SMA subjects was significantly reduced compared with the cells obtained from control subjects.

### Evaluations for comparison among SMA subjects

**Fig 5:**
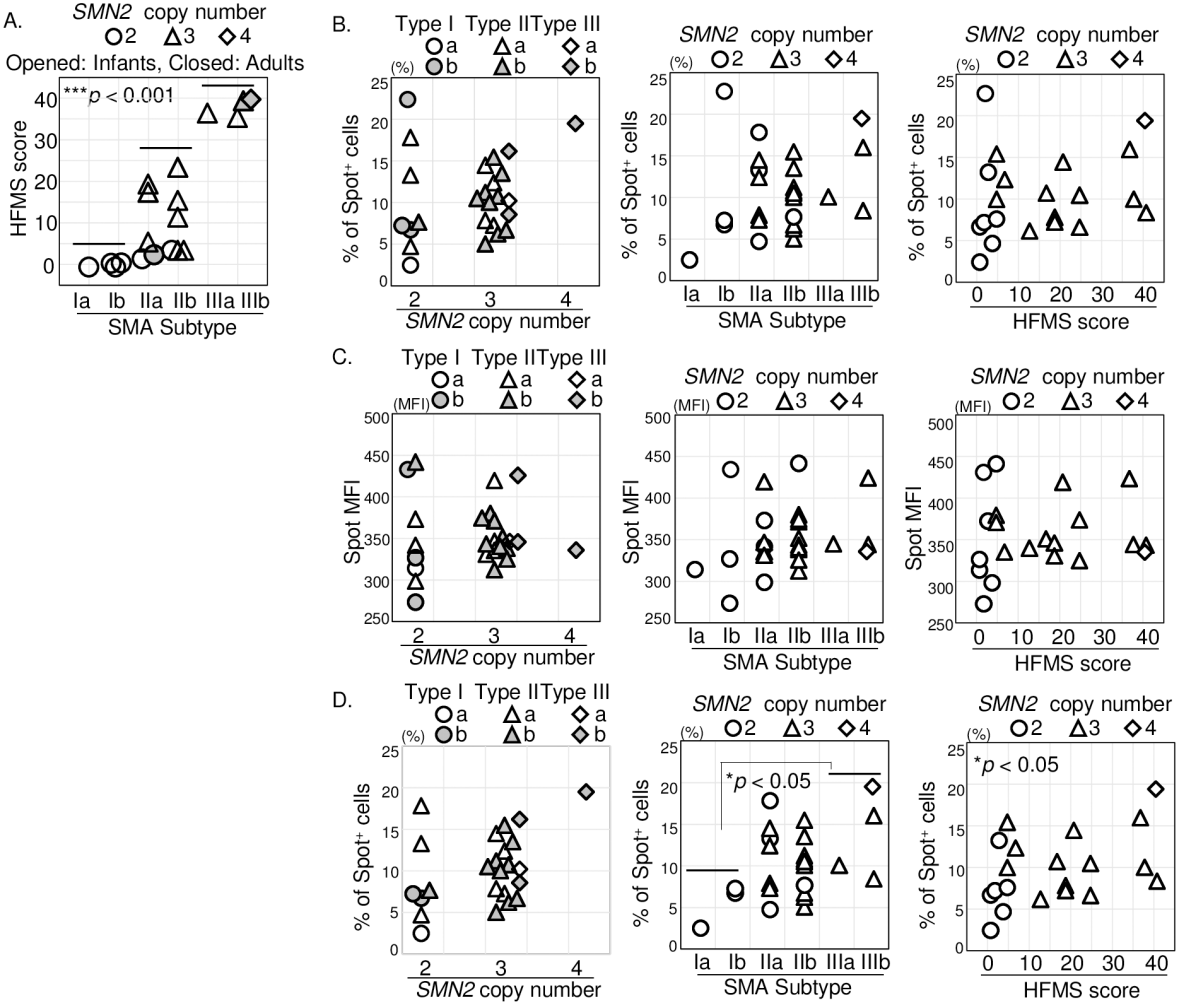
The correlation between survival motor neuron (SMN) protein expression among the spinal muscular atrophy (SMA) subjects. The analyses of SMN protein expressions. (A) The scatter diagram plotting correlation among the clinical subtype of SMA (x-axis), the score of HFMS (y-axis), and the copy number of *SMN2* (*symbols*) of the SMA subjects. Each symbol indicates that *SMN2* copy number is two copies (*circles, seven*), three copies (*triangles, thirteen*), and four copies (*diamonds, one*). Opened symbols are infant (*eighteen*) and closed symbols are adult (*three*) SMA subjects. (B) The percentage of SMN-spot^+^ cells and (C) the spot median fluorescence intensity (MFI) of CD33^++^ cells among 25 (or 21) SMA subjects. (B,C) The x-axis of the plots are the copy number of *SMN2* (*twenty-five*), the clinical subtype of SMA (*twenty-five*), and the score of HFMS (*twenty-one*). Each symbols are detailed on the top of plots. The copy number of *SMN2*, the clinical subtype of SMA, and the score of HFMS are provided in Table 1. The classification of clinical subtype of SMA is described in *materials and methods*. (D) The percentage of SMN-spot^+^ cells except for S2 (subject ID) from 25 (or 21) SMA subjects. Both the x-axis and the y-axis are shown in the same way as in (B). The correlation between % of spot^+^ cells and SMA subtype (I vs III); *rs* = 0.891, *p < 0.05. The correlation between % of spot^+^ cells and HFMS score; *rs* = 0.469, *p < 0.05. The numerical data of all subjects are provided in Table 1 and Supporting S4 Table online.

To evaluate the differences in the expression of SMN protein among SMA subjects, we focused on the analytical data generated from these twenty-five subjects (Fig 5). First, we confirmed the correlation among the *SMN2* copy number, the clinical subtype of SMA, and the scores of HFMS. As shown in Fig 5*A*, the scores of HFMS was significantly different depending on the *SMN2* copy number (*p* < 0.01, two copies versus three and four copies), and the clinical subtypes of SMA (*p* < 0.001); type I versus type II (*p* < 0.05), type II versus type III (*p* < 0.05), and type I versus type III (*p* < 0.001).

Since a marked significant difference was observed between control and SMA subjects in the case of SMN spot analysis (Figs 3*C* and 4*C*, and online Supporting S4 Table), we next compared the percentages of SMN-spot^+^ cells and SMN spot MFI in CD33^++^ cells on the basis of the *SMN2* copy number, the clinical subtypes of SMA, and the scores of HFMS. While comparing the SMA subjects, with two versus three and four copies of *SMN2*, there was no significant difference in the percentages of SMN-spot^+^ cells and SMN spot MFI in CD33^++^ cells. While drawing a comparison among the clinical subtypes of SMA, no significant difference in the percentages of SMN-spot^+^ cells and SMN spot MFI were found. In addition, in the trend test using the HFMS score, there was again no significant difference between the percentages of SMN-spot^+^ cells and the SMN spot MFI (Fig 5*B* and 5*C*).

In order to further verify this result, we confirmed the details regarding the health status of the twenty-five SMA subjects. The subject with ID number S2 had 1% atypical lymphocytes on the day of blood collection, and had gingivitis in the oral cavity. In addition, S2 had symptoms of gastroenteritis until two days before the visit. For the other subjects, there were no specific symptoms described in the medical records. Statistical processing, except for subject S2, is shown in Fig 5*D*. In the comparison among the clinical subtypes of SMA, a significant difference between type I and type III (*p* < 0.05) in the percentages of SMN-spot^+^ cells was observed. Furthermore, a significant difference was seen in the percentages of SMN-spot^+^ cells on the basis of the score of HFMS (*p* < 0.05) by Jonckheere-Terpstra trend test and Spearman’s rank correlation test. These data suggest that the percentage of SMN-spot^+^ cells seem likely to correlate with clinical subtype of SMA and the HFMS score when the SMA patient with the suspected infection was excluded.

### Co-staining of SMN and Smith core proteins in the nuclei

**Fig 6:**
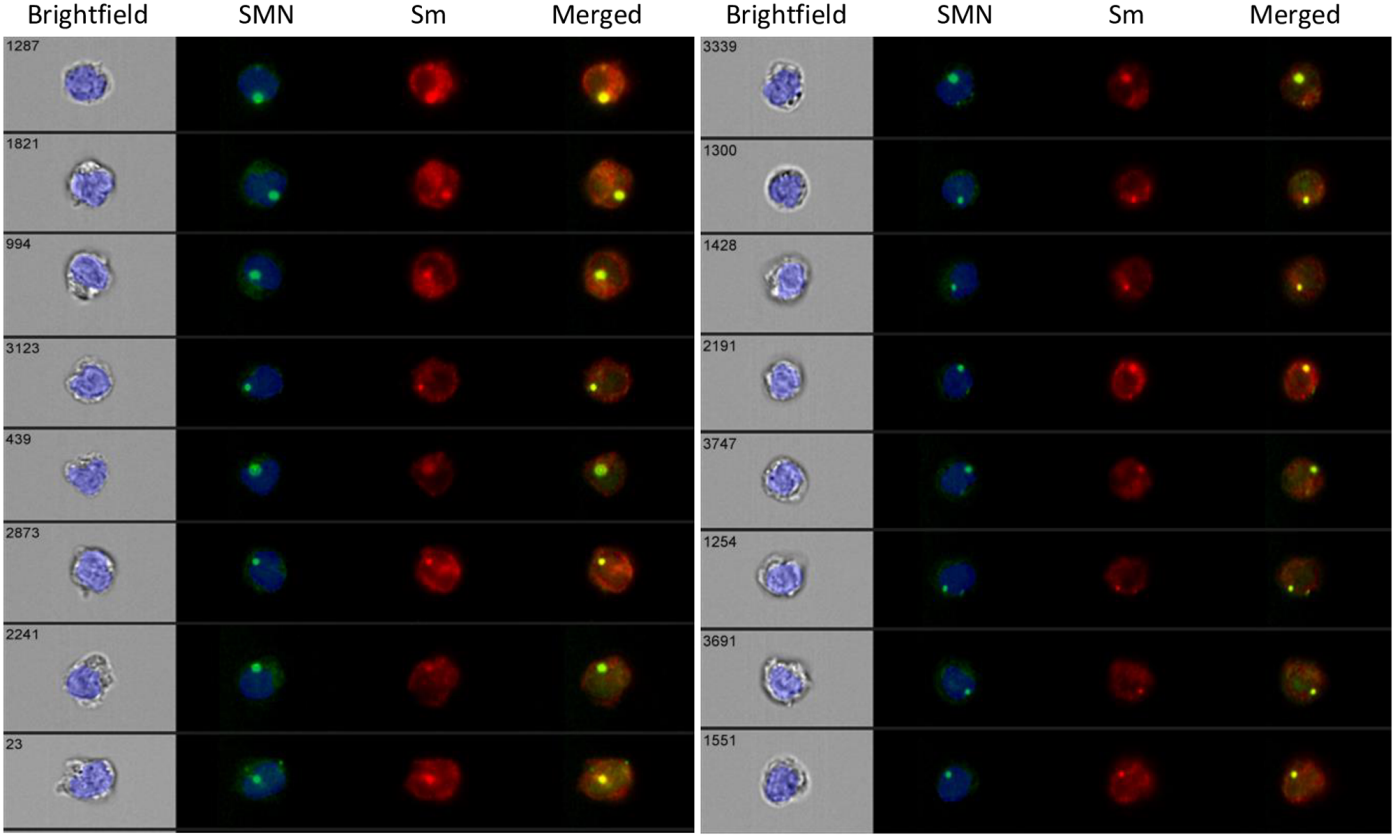
Localization of the survival motor neuron (SMN) proteins with Smith core proteins (Sm) in CD33^++^ cells. Cells were co-stained with 2B1 and anti-Sm mAb (Y12). The cells shown were extracted from SMN-spot^+^ CD33^++^ cells. Data were obtained from two representative individuals; control(C8, 7y; *left*) and SMA (S7, 5y, *right*) subjects among the eight examined. The eight-cells were extracted from the SMN spot with the highest intensity. The number in the upper left of the brightfield represents the individual number, depending on the detection order. Images are shown as bright field, nucleus (*blue*), SMN (*green*), Sm (*red*), and merged (*yellow*).

To confirm whether SMN protein was formed the complex in the CD33^++^ cells, we simultaneously examined staining with an antibody against Smith core proteins. As shown in Fig 6, SMN spots (*green*) and Smith core proteins spots (*red*) matched (*yellow*). These observations indicated that the co-localization of SMN protein and Smith core proteins in CD33^++^ cells.

## Discussion

### Application of imaging flow cytometry for semi-quantitative analysis

Several previous studies achieved quantification of the SMN protein from cells and tissues. Kolb *et al*. [17] developed an immunoassay-based method using chemiluminescent detection in peripheral blood mononuclear cells (PBMCs). Crawford *et al*. [7] and Kobayashi *et al*. [18] devised a sandwich ELISA method using PBMCs. Makhortova *et al*. [19] established a quantitative method using immunostaining and confocal fluorescence microscopy. Naryshkin *et al*. [20] demonstrated homogeneous time-resolved fluorescence resonance energy transfer, employing animal tissues and human fibroblasts. Steinkellner *et al*. [21] and Czech *et al*. [8] established the Elecsys^®^ platform’s ECLIA method using buccal cells or whole blood. Zaworski *et al*. [22] used the Meso Scale Discovery^®^ platform’s electrochemiluminescence (MSD-ECL) with whole blood.

In the present study, we developed a method to evaluate the expression of SMN protein in PBCs using imaging flow cytometry. The differences between previous approaches [7, 8, 17–22] and our method are described as follows: First, we separately analyzed levels of SMN protein expression in cells classified according to four typical types of PBCs, as the levels of SMN protein expression markedly differed among the cell types. In addition, the proportion of cell types in PBCs varies depending on physical conditions, particularly in infants. Second, we analyzed fixed rather than lysed cells, to avoid the degradation of the SMN protein by released proteases or chemical substances from lysed cells. The analytical methods used for the quantification of human SMN protein in recent studies are summarized online in Supporting S5 Table.

The relative comparison of protein levels was conducted using imaging flow cytometry, which is more precise than conventional flow cytometry. Among the advantages of our system is the high sensitivity in the detection of fluorescence by a mounted “time-delay integration charge-coupled device” (TDI-CCD) camera. In addition, the analytical software used in our system provided flexibility and control of settings, including various models of the algorithm for the scaled images (features), and definition of the regions of interest (masks) by applying an image of a single cell. Moreover, the visualization system for imaging flow cytometry is provided as “logicle” scaling, which is different from typical logarithmic scaling used in conventional flow cytometry. The method of “logicle” scaling display achieves a more appropriate analysis, for expansion of the dynamic range and compensation for fluorescence-interference, when compared to the logarithmic scaling display method [30].

These characteristic advantages of imaging flow cytometry allowed us to perform semi-quantitative analysis of SMN protein using only 1.5 mL of peripheral blood. Since the cells were prepared employing erythrocyte hemolysis, it was possible to minimize cell loss. Freshly collected blood samples could be left at room temperature for up to 24 h with no critical influences. Consequently, blood samples could be transported from multiple clinical sites to the research laboratory within a day.

### Spot analysis of SMN protein in CD33^++^ cells

Although the expressions of SMN protein in CD33^++^ cells varied widely among the data from individual control subjects, these variations were limited among the individuals with SMA (Figs 3 and 4). In fact, when we attempted to compare the standard deviations (SD) for the five evaluated items that showed significant differences between control and SMA subjects in CD33^++^ cells (*p* < 0.001, see online Supporting S6 Table), the SD of all evaluations from SMA subjects was lesser than that of the control subjects (SMA SD < control SD). In particular, the results of SMN spot analysis (*right*, two columns) were more remarkable than the cellular MFI (*left*, two columns) of the SMN protein in CD33^++^ cells (SMA SD: control SD ratio = 0.38, 0.59). Since the genetic background of SMA subjects is restricted by SMN1 deficiency, the SD of analytical values is limited. In the case of control subjects, analytical values are not limited, because the genetic background of them is not restricted. We then speculated about the result of the spot analysis. SMN spot analysis revealed an localization of SMN protein. This protein localization may imply the oligomerization of the SMN protein. In addition, SMN proteins from SMA subjects contained SMNΔ7 or other mutated forms of the protein that are unstable and show defective oligomerization [5, 6]. The complex formation and functional biogenesis of the SMN protein in SMA subjects would presumably be reduced.

Furthermore, as the result of the validation test of analysis methods shown in online Supporting S3 Table, the RSD value in the reproducibility of ΔMFI was more than 10% (18.8%–40.2%), while that of the percentage of spot^+^ cells was within 10%. This indicated the validity of the spot analysis. The examination of intracellular or intranuclear ΔMFI must subtract the autofluorescence intensity of stained cells by isotype control. Contrary to this detail, the spot analysis made it possible to compare between the SMN stained cells, without the need of considering the autofluorescence intensities of the isotype control stained cells. Because the autofluorescence intensities of the cells did not influence spot analysis (see online Supporting S3*E* Fig (R4), spot^+^ cells < 1.0%), SMN spot MFI could be assumed to reflect the degree of functional SMN localization. Hence, we thought that the spot analysis was considered to be more properly reflect the functional SMN than MFI comparison.

In addition to those in CD33^++^ cells, SMN spots in CD3^+^ T cells, CD19^+^ B cells, and CD33^+^ neutrophils were also analyzed. We concluded that our SMN spot analysis method was not appropriate for the populations other than CD33^++^ cells. However, the level of systemic SMN protein showed a correlation between the SMA group and control group for both CD3^+^ T cells, and CD19^+^ B cells (Fig 3*A*, 3*B* and 3*C*). Further details are discussed in online Supporting discussion (S1 Text).

### Identification of functional SMN protein

In recent years, SMN has been shown to function as a component of a protein complex that includes Gemin components [31], Smith core proteins [32, 33], and other interacting factors [34, 35] have been investigated. All previous studies of SMN complexes, involving frozen tissues [36], or cultured cells [29] employed combinations with immunoprecipitation or immunocytochemical staining. We attempted to examine whether the SMN spots in CD33^++^ cells reflected formation of the functional complex with nuclear foci in peripheral nuclear cells. As mAb 2B1, which is specific for SMN protein, recognizes the epitope alignment of seven amino acids in exon 1 of SMN protein, our method could detect various forms of the SMN protein, including SMNΔ7. In addition, the binding of 2B1 should exert little influence on the functional associations of SMN protein, such as oligomerization [5], the binding to Smith core proteins [37, 38], and the binding to Gemin 2 [39, 40]. However, the localization of SMN protein would be uniformly reduced in Type I, Type II, and Type III SMA, for reasons described above (Fig 4). As shown in Fig 6, we succeeded the co-staining of Smith core proteins with the SMN protein. Thus, SMN spots present in the nucleus may form functional complexes in CD33^++^ cells. To our knowledge, these results are the first to demonstrate the presence of functional SMN protein in freshly isolated PBCs.

### Possibilities of “heterogeneity” and “plasticity” of monocytes

Although SMA is caused by the degeneration of alpha motor neurons in the anterior horn of the spinal cord, the reason that CD33^++^ cells in peripheral blood can be used as an index for the evaluation of SMN protein remains unknown. Based on our results, we can speculate about the possible reasons. The population of CD33^++^ cells contained 85%–90% CD14^+^ monocytes and 4%–6% lineage marker^−^ (Lin^−^) cells (data not shown). With almost 90% of the CD33^++^ cells being CD14^+^ monocytes, SMN-spot^+^ CD14^+^ monocytes would be the predominant cell type among SMN-spot^+^ CD33^++^ cells.

Circulating monocytes originate from hematopoietic stem cells (HSCs) that serve as progenitor cells with the capability of further differentiation [41]. Recent understanding of circulating monocytes suggests that there are two principal subsets. One monocyte subset progresses to patrol intravascular organs, whereas the other subset can exit the blood stream to patrol extravascular organs, and subsequently differentiate into organ-specific macrophages [42]. The latter includes macrophages, such as those located in the dermis, Peyer’s patches, heart, kidney, and lymphoid organs. These circulating monocyte-derived macrophages have the capacity of proliferating the tissue, and transporting antigens to regional lymph nodes during inflammation. In addition, Langerhans cells, microglia cells, Kupffer cells, osteoclasts, alveolar macrophages, and peritoneal macrophages can all differentiate from circulating monocytes in vitro, although fate mapping of these cells revealed that they were not derived from circulating monocytes in vivo [41–43]. Despite a small cell population in PBCs, monocytes are crucial cells that have the potential to differentiate into diverse phenotypes of the macrophage lineage and to be systemically distributed.

On the other hand, monocytes are derived from bone marrow HSCs that are of mesodermal origin, and neural cells are derived from ectodermal progenitors. In recent years, several cultured human-cell populations originating from circulating monocytes have been reported. Those cultured monocytes have the capacity to differentiate into non-phagocytic pluripotent stem cell-like phenotypes and are named “pluripotent stem cells (PSCs)” [44], “monocyte-derived multipotential cells (MOMCs)” [45–47], or “programmable cells of monocytic origin (PCMOs)” [48, 49]. As proof of pluripotency, cultured monocytes are reported to differentiate into epithelial-, endothelial-, hepatocytic-, pancreatic islet-, and neuronal-phenotype cells. The capacities of these pluripotent cells have been demonstrated to include differentiation into both mesodermal and ectodermal lineages, similar to mesenchymal stem cells.

According to these investigations, CD14^+^ monocytes included among CD33^++^ cells, are characterized as demonstrating “heterogeneity” and “plasticity”. These CD14^+^ monocytes are assumed to be in a slightly activated state circulating in blood vessels in order to play a role in the innate immune response [50] and further differentiation [51, 52]. Moreover, to maintain biological homeostasis, it is assumed that a similar system exists in the biogenesis or intracellular trafficking of SMN among somatic cells, despite differences between neural cells and bone marrow cells in the developmental processes of differentiation [53]. At present, there is no reliable argument but, rather, only anticipation. Elucidating the underlying mechanism(s) is expected to allow novel interpretations of the advancement of our research in the future.

### The points to be improved for estimation of functional SMN protein

As discussed above, these findings allowed us to identify the relationship between the pathological conditions and the systemic levels of functional SMN protein in individuals with SMA (Fig 5). In particular, the correlation between the evaluation of physical ability and functional SMN was expected. There were two patients (S1 and S4) whose motor skills were very limited and who both had scores of 0 on the HFMS. In the evaluation by CHOP INTEND, S1 scored 23 (type Ia) and S4 scored 35 (type Ib) out of full scores of 64. In parallel, the percentages of SMN spot^+^ cells in S1 and S4 were 2.3% and 7.0%, respectively. We anticipate that SMN spot analysis will become the primary endpoint for monitoring therapeutic intervention, serving as a biological marker of SMA therapeutic efficacy [24].

However, we must mention exceptions to the analytical values considered among the twenty-five SMA subjects. Patient S2 whose data was excluded from the statistical analysis was a SMA patient suspected to have an unidentified infection because of detection of an atypical lymphocyte profile. At the same time, gingivitis and gastroenteritis were also observed as health factors for S2. The percentage of SMN-spot^+^ cell in the sample from S2 showed the highest value among the twenty-five SMA subjects at 23.2%, (online Supporting S4 Table). It was predicted that systemic immune responses would be activated because of innate immunity induced by bacteria or viruses from the oral cavity or gastrointestinal tract. It may be that granulocytes and monocytes were activated by the infection, resulting in the percentage of spot positive cells to increase [54, 55]. If the spot analysis is vulnerable to being affected by infections in the donor subjects, it will be necessary to consider what is required to maintain the accuracy of the analysis as a biomarker. Meanwhile, although we observed significant differences in the percentages of SMN spot^+^ cells based on clinical subtype of SMA and the score of HFMS, we could not detect clear differences on the basis of the copy number of *SMN2*. Moreover, despite the most marked difference obtained being the comparison of the SMA subjects with the control subjects, no clear difference was detected in the MFI of SMN spot based on the HFMS score. This outcome is presumed to be due to two reasons. First, since there was little variation in the data among SMA subjects, it is difficult to obtain a significant difference. As mentioned in the section on “*Spot analysis of SMN protein in CD33^++^ cells*” the SD of all evaluations of the SMA subjects was lesser than that of the control subjects (SMA SD < control SD) (online Supporting S6 Table). It is possible that the correlation between SMN spot analysis and the clinical condition was lost due to individual variability seen among the SMA subjects. Therefore, it will be necessary to examine more SMA subjects in order to have a larger sample base. Next, and most importantly, a more accurate definition of SMN spot analysis is required. Lefebvre *et al*. [56], Boulisfane *et al*. [57], and Tapia *et al*. [58] demonstrated that reduced expression of the SMN protein in SMA is also associated with a failure of the SMN complexes to assemble into nuclear bodies. Shpargel *et al*. [33] observed defective SMN assembly in Type I, but not in Type III SMA with mutated recombinant SMN proteins. In subsequent work, we must examine the association between the co-localization of SMN as a functional molecule in CD33^++^ cells and its clinical pathology. It is necessary to construct a more accurate spot detection algorithm. In addition, it is also important to generalize this analysis method. We will attempt to clarify the correlation between the pathological severity of SMA and functional SMN protein in peripheral blood cells using effective imaging variables. Essentially, in order to gain the information useful for developing SMA therapies, we must investigate the physiological events in greater detail, focusing on the monitoring of SMN-behavior as a reflection of the therapeutic efficacy in SMA.

## Conclusion

We have developed a method allowing the evaluation of the functional SMN protein in PBCs using imaging flow cytometry. The expression of SMN protein in CD3^+^, CD19^+^, and CD33^++^ cells obtained from SMA patients, was significantly reduced compared with that in cells from control subjects. Notably, in spot analysis of CD33^++^ cells, the intensities of SMN spots were significantly reduced in SMA subjects, when compared with that in controls. Moreover, the defined SMN spots were co-localized with Smith core proteins. Therefore, SMN spots implied the presence of functional SMN protein in CD33^++^ cells. We anticipate that SMN spot analysis will become the primary endpoint assay for the evaluation and monitoring of therapeutic intervention, with SMN serving as a reliable biomarker of therapeutic efficacy in SMA patients.

## Supporting information

S1 Fig Procedure for the selection of target cells prior to analysis of four populations of peripheral blood nuclear cells (PBCs). (PDF)

S2 Fig Details of the algorithm designed for detecting survival motor neuron (SMN) spots in CD33^++^ cells. (PDF)

S3 Fig Survival motor neuron (SMN) staining image of four populations in peripheral blood nuclear cells (PBCs). (PDF)

S1 Text Spot analysis of SMN protein in CD3^+^ T cells, CD19^+^ B cells, and CD33^+^ neutrophils. (Discussion).
(PDF)

S1 Table Cell surface phenotype of peripheral blood cells (PBCs) population. (PDF)

S2 Table Verification of imaging flow cytometry (IFC) for the semi-quantitative analysis. (PDF)

S3 Table Verification of the reproducibility of the SMN spot-detection method. (PDF)

S4 Table Raw data of median fluorescence intensity (MFI) and spot analysis in CD33^++^ cells. (PDF)

S5 Table Summary of the analytical methods for the quantification of the human SMN protein. (PDF)

S6 Table The variations in the standard deviation (SD) of the evaluation items with significant differences. (PDF)
