# Supplementary material for: A new biomarker candidate for spinal muscular atrophy: Identification of a peripheral blood cell population capable of monitoring the level of survival motor neuron protein
Source: PLoS One. 2018 Aug 13;13(8):e0201764. doi: 10.1371/journal.pone.0201764 (PMC6089418; doi:10.1371/journal.pone.0201764)
Supplement: S1 Fig — (PDF) [file pone.0201764.s001.pdf]

Supporting information
Figure S1

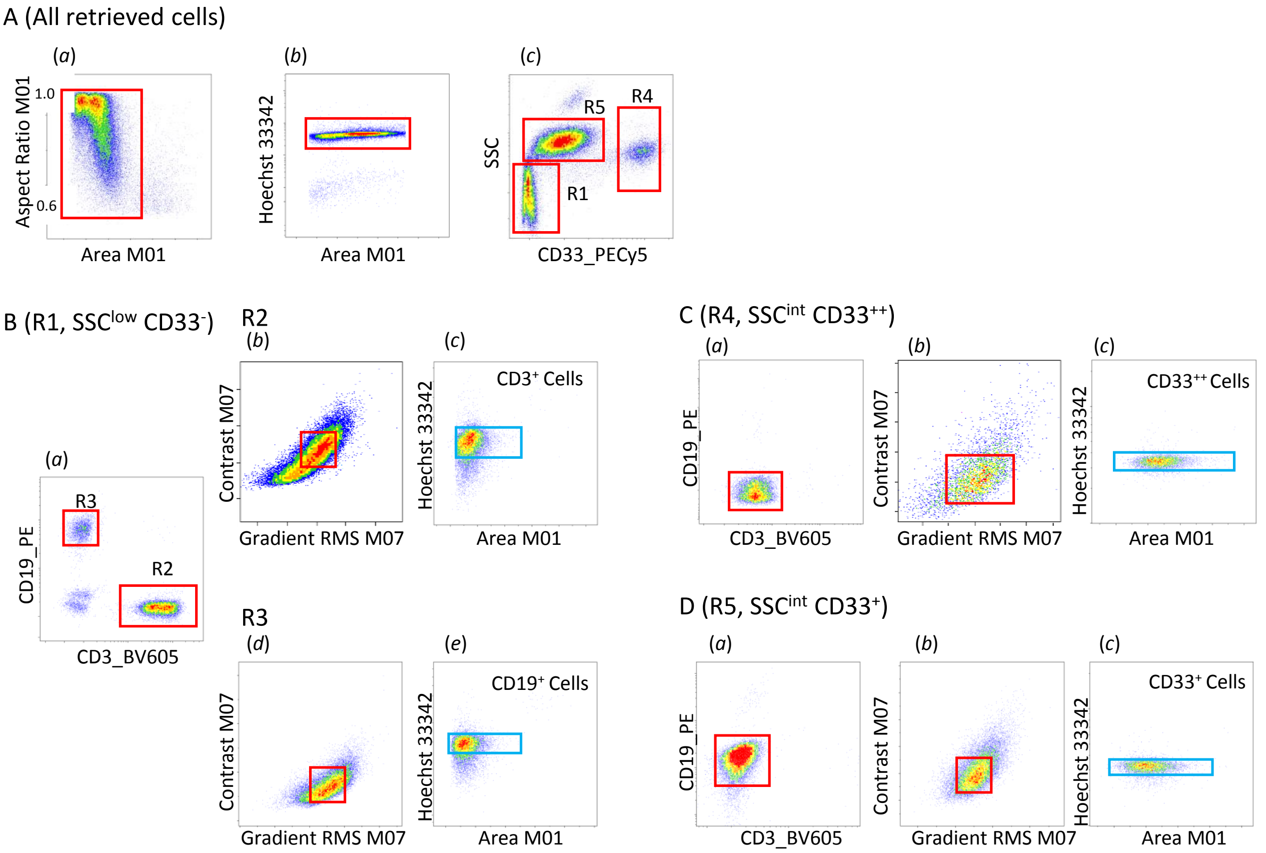

Figure S1
Procedure for the selection of target cells prior to analysis of four populations of peripheral blood nuclear cells (PBCs)

(A) For all retrieved cells. (*a*) PBCs were roughly gated to exclude aggregated cells. Cells were analyzed for “Area” (x-axis) and “Aspect ratio” (y-axis), as plotted in the bright field (Ch01). The “Area” feature indicates cell size, whereas the “Aspect ratio” indicates the ratio of the minor axis to the major axis (Area Ch01: 60–195 pixels, Aspect ratio Ch01 > 0.6). (*b*) Nuclear cells were gated by exclusion of erythrocytes and debris. Cells were analyzed for “Area” (x-axis) and Hoechst 33342 (y-axis). (*c*) Single-nuclear cells were analyzed using parameters of CD33 (x-axis) and side scatter (SCC, y-axis). Cells were roughly divided into three populations: SSC^{low} CD33$^{–}$, SSC^{int} CD33^{+}, and SSC^{int} CD33^{++} cells.
(B) For SSC^{low} CD33$^{–}$ cells (R1). (*a*) SSC^{low} CD33$^{–}$ cells were analyzed by CD3 (x-axis) and CD19 (y-axis). CD3^{+} CD19$^{–}$ cells (R2) are displayed in (*b*) and (*c*). CD19^{+} CD3$^{–}$ cells (R3) are displayed in (*d*) and (*e*).
Gated CD3^{+} cells (*b*) or CD19^{+} cells (*d*) are displayed in a plot with “Gradient RMS” (x-axis) and “Contrast” (y-axis) in the Ch07 based on Hoechst intensity. These plots are useful for determining focus quality in the nucleus. The next plots, focused CD3^{+} cells (*c*) and CD19^{+} cells (*e*) are displayed with “Area” Ch01 (x-axis) and Hoechst 33342 (y-axis) to exclude doublet cells and to select similar Hoechst intensities for all target cells.
(C) For SSC^{int} CD33^{++} cells (R4). (*a*) Extracted SSC^{int} CD33^{++} cells in A (*c*) were used to develop a plot using CD3$^{–}$ (x-axis) and CD19$^{–}$ (y-axis) to exclude other types of cells. The plots of (*b*) and (*c*) are displayed using the same axes as those described in B.
(D) For SSC^{int} CD33^{+} cells (R5). (*a*) Extracted SSC^{int} CD33^{+} cells in A (*c*) were used to develop a plot using CD3$^{–}$ (x-axis) and CD19$^{–}$ (y-axis) to exclude other types of cells. The plots of (*b*) and (*c*) are displayed using the same axes as those described in B.
