# Supplementary material for: A new biomarker candidate for spinal muscular atrophy: Identification of a peripheral blood cell population capable of monitoring the level of survival motor neuron protein
Source: PLoS One. 2018 Aug 13;13(8):e0201764. doi: 10.1371/journal.pone.0201764 (PMC6089418; doi:10.1371/journal.pone.0201764)
Supplement: S2 Fig — (PDF) [file pone.0201764.s002.pdf]

Supporting information
Figure S2

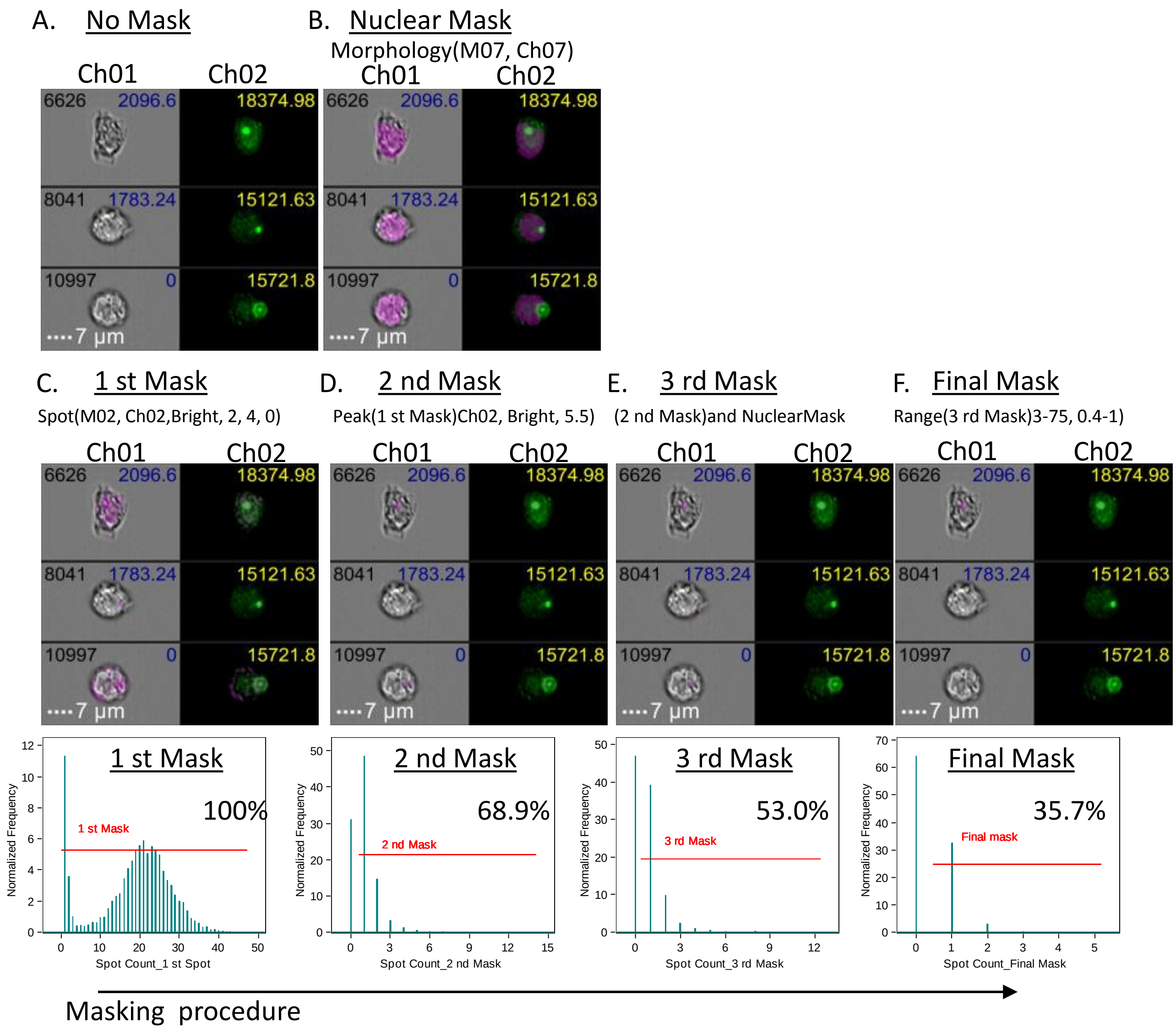

Figure S2

Details of the algorithm designed for detecting survival motor neuron (SMN) spots in $CD33^{++}$ cells.

In the imaging panels, three cells were shown in the bright field Ch01 (*left*) and Ch02 (*right*) representative of $CD33^{++}$ cells in control subjects (C7). The number displayed at the top left of the cell in Ch01 (*black*) represents the order of detection, that at the top right of the cells in Ch01 (*blue*) represents the fluorescence intensity (FI) in the limited SMN spot area (SMN spot FI), and that at the top right of cells in Ch02 (*yellow*) represents the FI of SMN in the whole cell (Intracellular SMN FI) of single cell. No. 6626 and No. 8041 cells were SMN-spot^{+} cells (upper two cells, FI = 2096.6, 1783.2). The No. 10997 cell was a SMN-spot^{-} cell (lowest, FI = 0).

(A) Cells are shown without a mask both bright field (Ch01) and AF488-2B1 (Ch02).
(B) Cells are shown with an overlaid nuclear mask (violet) both bright field (Ch01) and AF488-2B1 (Ch02).

(C–F) The procedure for detecting the SMN spot is shown. The overlaid violet area is shown as the defined spot mask in each process of the masking procedure.
(C) The “Spot mask” in Ch02 was used as the first mask. The percentage of selected cells through the first mask was 100%.
(D) The detected spots through the first mask were defined according to the “Peak mask” in Ch02 (second mask). The percentage of selected cells through the second mask was 68.9%.
(E) The detected spots through the second mask were limited to the nuclear area, as already defined with the “Morphology mask” in Ch07 (third mask). The percentage of selected cells through the third mask was 53.0%.
(F) The detected spot through the third mask was defined with the “Range mask” in Ch02 (Final mask). The percentage of selected cells through the final mask was 35.7%.
Histograms under the panels indicate the percentage of spot-positive cells (y-axis) and the spot count (x-axis).
