# Supplementary material for: A new biomarker candidate for spinal muscular atrophy: Identification of a peripheral blood cell population capable of monitoring the level of survival motor neuron protein
Source: PLoS One. 2018 Aug 13;13(8):e0201764. doi: 10.1371/journal.pone.0201764 (PMC6089418; doi:10.1371/journal.pone.0201764)
Supplement: S3 Fig — (PDF) [file pone.0201764.s003.pdf]

Supporting information
Figure S3

A. CD3⁺ Cells (R2)

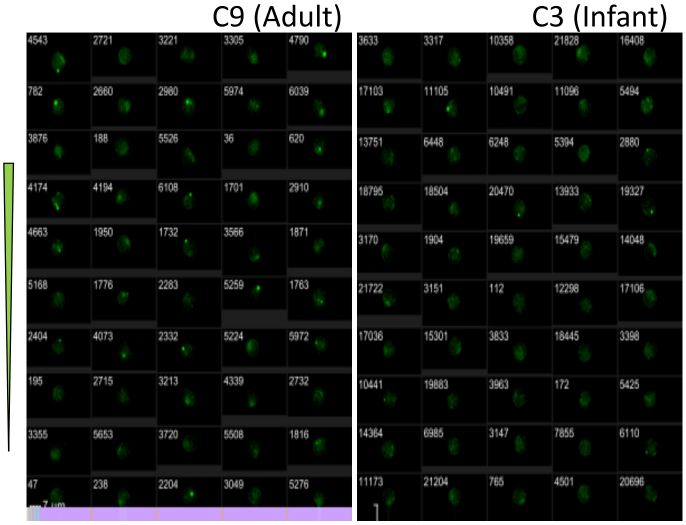

B. CD19⁺ Cells (R3)

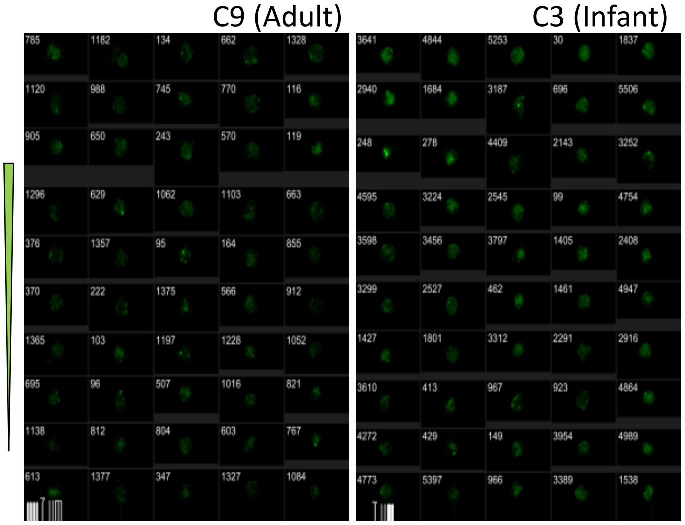

C. CD33⁺⁺ Cells (R4)

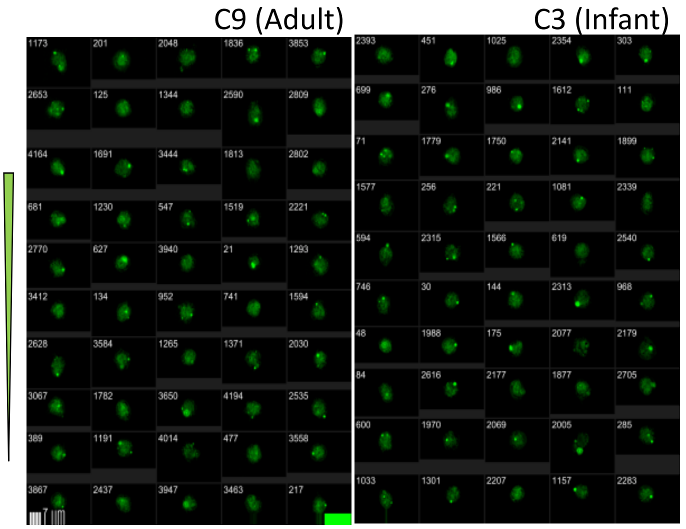

D. CD33⁺ Cells (R5)

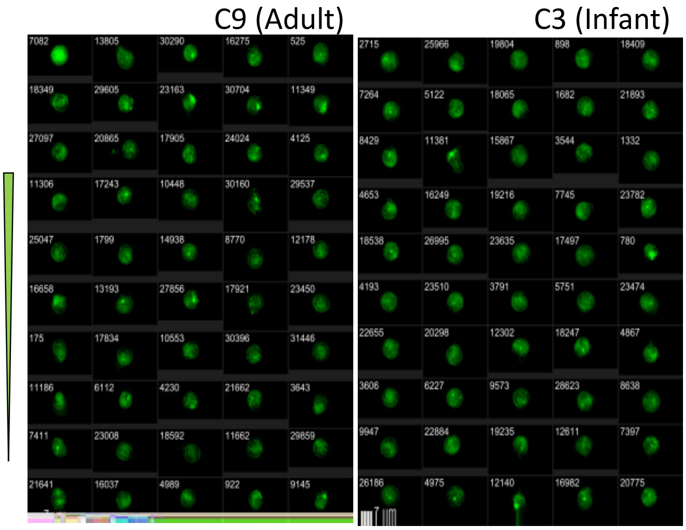

E. Percentage of non-specific spot⁺ cells

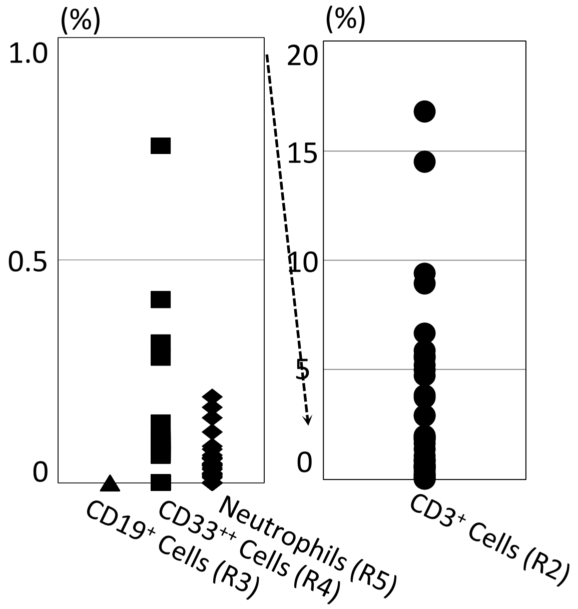

F.

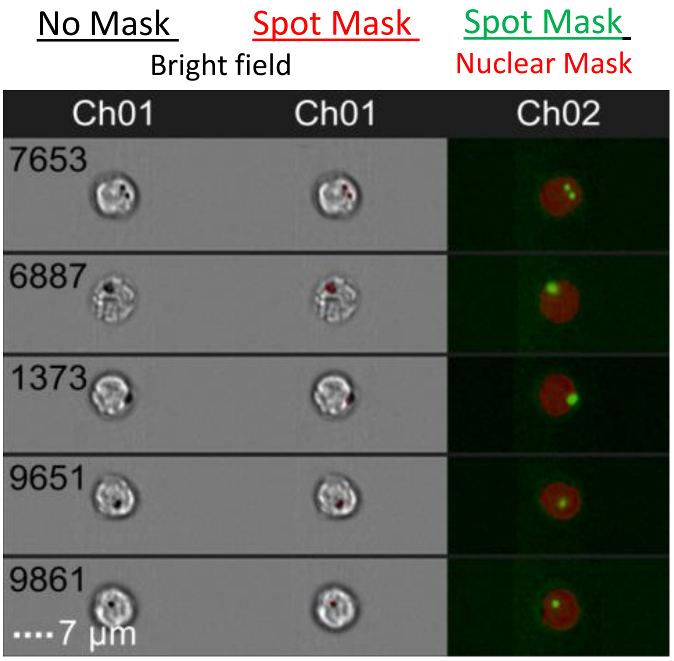

Figure S3

Survival motor neuron (SMN) staining image of four populations in peripheral blood nuclear cells (PBCs).

SMN staining image in (A) $CD3^+$ cells (R2), (B) $CD19^+$ cells (R3), (C) $CD33^{++}$ cells (R4), and (D) $CD33^+$ cells (R5). Data shown in the panels represent an adult and an infant control subject (C9, C3). In total, 50 cells were extracted from the highest intensity of the median fluorescence intensity (MFI) order. The image panels are shown at the same level on the brightness setting.

(E) Percentage of spot-positive cells ($spot^+$ cells) in each population stained with AF488-MOPC21 as an isotype control IgG (all 25 subjects). (*left*) The maximum percentage of nonspecific $spot^+$ cells in $CD19^+$ cells (R3), $CD33^{++}$ cells (R4), and neutrophils (R5) is < 1.0%. (*right*) The maximum percentage of nonspecific $spot^+$ cells in $CD3^+$cells (R2) is >15%.

(F) Imaging analyzer-detected nonspecific spots in $CD3^+$ cells. $CD3^+$ cells were stained with AF488-MOPC21. The spot-detecting cells (Ch02) were observed under the bright field (Ch01) of view. (*right*) Spot-detecting cells under the Ch02 are shown. Detecting spot are indicated by green color. The overlaid red area represents the nuclear mask.

(*middle*) Spot-detecting cells under the bright field are shown. Detecting spot (same as right panel) are indicated by red color.

(*left*) Spot-detecting cells under the bright field are shown. No mask was overlaid.
