# Supplementary material for: A new biomarker candidate for spinal muscular atrophy: Identification of a peripheral blood cell population capable of monitoring the level of survival motor neuron protein
Source: PLoS One. 2018 Aug 13;13(8):e0201764. doi: 10.1371/journal.pone.0201764 (PMC6089418; doi:10.1371/journal.pone.0201764)
Supplement: S1 Text — (Discussion). (PDF) [file pone.0201764.s004.pdf]

Supporting Text

Discussion

Spot analysis of SMN protein in CD3$^+$ T cells, CD19$^+$B cells, and CD33$^+$ neutrophils

Besides CD33^{++} cells, SMN spots in CD3$^+$ T cells, CD19$^+$ B cells, and CD33$^+$ neutrophils were also analyzed. In CD3$^+$ T and CD19$^+$ B cells, SMN proteins were not punctate (Figure 3*C*, R2 and R3), but rather diffusely distributed in the cytoplasm and nucleus (Figure 3*AB*, R2 and R3). Generally, the localization of the SMN protein complex in nuclear foci has been assumed due to depending on cell cycle progression. CD3$^+$ T and CD19$^+$ B cells are the typical immunocompetent cells in acquired immunity. As immune responses were not elicited against antigens, freshly isolated CD3$^+$ T and CD19$^+$ B cells were in a steady state in the cell cycle. Therefore, diffusely stained SMN proteins in both CD3$^+$ T and CD19$^+$ B cells would presumably reflect the level of systemic SMN proteins (Figure 3*ABC*, and Supporting Figure S3 online).

In addition, IgG aggregation has been detected in CD3$^+$ T cells stained with isotype control IgG (0%–16.7%, Supporting Figure S3*E* online). According to observation in the bright field of view, these nonspecific spots were presumed to be due to the cytoplasmic structure in T cells (Supplementary Figure S3*F* online). Thus, we concluded that the spots detected in T cells must have contained the nonspecific IgG aggregations. Interestingly, such a structure was not observed in B cells.

In neutrophils, SMN spots were detected in a broad range of cell percentages (3.0%–82.1%) in both groups of the control and SMA subjects (Figure 3*C*, R5). Nonspecific aggregations of isotype control IgG in neutrophils were very few (mean; 0.03%, Supplementary Figure S3*E* online), whereas we could not estimate whether the detected spots were SMN-specific. Neutrophils were derived from common myeloid progenitor cells of HSCs origin, as with monocytes and dendritic cells. However, we could not find an appropriate method of analyzing images by focusing on neutrophils with complicated shapes, including the intricate structures of cells, the segmented nucleus, intracellular granules, and non-uniform shapes. In addition, neutrophils have the highest autofluorescence background among peripheral blood nuclear cells. Therefore, we may not be able to analyze the SMN protein level accurately in R5-gated neutrophils. Meanwhile, the activation state of neutrophils is readily influenced by the physical conditions of subjects, such as infectious diseases, fever, and injury. For all these reasons, we determined that neutrophils are an unsuitable cell population to serve as an index for the evaluation of SMN protein.
