# Supplementary material for: A new biomarker candidate for spinal muscular atrophy: Identification of a peripheral blood cell population capable of monitoring the level of survival motor neuron protein
Source: PLoS One. 2018 Aug 13;13(8):e0201764. doi: 10.1371/journal.pone.0201764 (PMC6089418; doi:10.1371/journal.pone.0201764)
Supplement: S1 Table — (PDF) [file pone.0201764.s005.pdf]

Supporting Information, Table S1

Table S1 Cell surface phenotype of peripheral blood cell (PBC) population

		SSC	CD33	CD3	CD19	CD45	CD14	HLA-DR	CD11c	CD15	CD66abce	Major population
R1	R2	Low	-	+	-	++	-	-	*n.d.*[a)]	-	-	T cells
	R3	Low	-	-	+	++	-	dim/+	*n.d.*	-	-	B cells
R4		Intermediate	++	-	-	+/++	dim/+	dim/++	+/++	+/-	+/-	Monocytes
R5		Intermediate	+	-	-	+	-	-	*n.d.*	++	++	Neutrophils

a) n.d.; not determined
