# Supplementary material for: A new biomarker candidate for spinal muscular atrophy: Identification of a peripheral blood cell population capable of monitoring the level of survival motor neuron protein
Source: PLoS One. 2018 Aug 13;13(8):e0201764. doi: 10.1371/journal.pone.0201764 (PMC6089418; doi:10.1371/journal.pone.0201764)
Supplement: S2 Table — (PDF) [file pone.0201764.s006.pdf]

Supporting Information, Table S2

Table S2 Verification of Imaging flow cytometry (IFC) for the semi-quantitative analysis.

IgG concentration (ng/ml)	0	11.2	22.3	44.6	89.3	178.6	*y=ax+b*	
							a (Slope)	r^2
Exp. 1	845	2072	3323	5957	11082	21581	116.5	1.000
Exp. 2	799	1999	3213	5688	10665	21142	114.4	0.999
Exp. 3	812	1936	3103	5476	10349	20770	112.6	0.999
Exp. 4	815	1970	3162	5600	10521	20860	112.9	0.999
Exp. 5	805	1893	3002	5250	10038	20341	110.4	0.999
Mean	815	1974	3161	5594	10531	20939	113	
SD	17.9	67.2	120.0	261.1	386.7	459.8	2.3	
CV (%)	2.2	3.4	3.8	4.7	3.7	2.2	2.0	
