# Supplementary material for: A new biomarker candidate for spinal muscular atrophy: Identification of a peripheral blood cell population capable of monitoring the level of survival motor neuron protein
Source: PLoS One. 2018 Aug 13;13(8):e0201764. doi: 10.1371/journal.pone.0201764 (PMC6089418; doi:10.1371/journal.pone.0201764)
Supplement: S3 Table — (PDF) [file pone.0201764.s007.pdf]

Supporting Information, Table S3

Table S3 Verification of the reproducibility of the SMN spot-detection method.

C15

	Cell count	Fluorescence intensity: CD33^{++} Cells Cell ΔMFI	Fluorescence intensity: CD33^{++} Cells Nuclei ΔMFI	Fluorescence intensity: Spot^{+} cells Nuclei MFI	SMN spot analysis: SMN spot MFI	SMN spot analysis: Percentage of spot^{+} cells
Exp. 1	2375	7594	4502	8998	541	33.1
Exp. 2	2578	4555	2878	7416	434	37.4
Exp. 3	2707	5666	3510	8073	470	32.2
Exp. 4	2876	4504	2828	7437	430	36.0
Exp. 5	2333	8826	5359	10018	578	36.7
Mean		6229	3815	8388	490	35.1
SD		1917	1096	1115	66	2.3
RSD (%)		30.8	28.7	13.3	13.5	6.6

C16

	Cell count	Fluorescence intensity: CD33^{++} Cells Cell ΔMFI	Fluorescence intensity: CD33^{++} Cells Nuclei ΔMFI	Fluorescence intensity: Spot^{+} cells Nuclei MFI	SMN spot analysis: SMN spot MFI	SMN spot analysis: Percentage of spot^{+} cells
Exp. 1	2212	6894	4995	8529	606	43.5
Exp. 2	2248	7051	5166	9230	607	48.6
Exp. 3	2951	6495	4942	9157	537	46.8
Exp. 4	3226	3566	1596	7921	641	39.4
Exp. 5	2531	4776	2993	7835	496	40.4
Mean		5756	3938	8534	577	43.5
SD		1523	1582	639	59	3.8
RSD (%)		26.5	40.2	7.7	10.2	8.7

C17

	Cell count	Fluorescence intensity: CD33^{++} Cells Cell ΔMFI	Fluorescence intensity: CD33^{++} Cells Nuclei ΔMFI	Fluorescence intensity: Spot^{+} cells Nuclei MFI	SMN spot analysis: SMN spot MFI	SMN spot analysis: Percentage of spot^{+} cells
Exp. 1	2864	5900	3611	7181	318	15.4
Exp. 2	2537	6223	3788	7634	291	15.8
Exp. 3	2816	5063	3008	6902	295	12.1
Exp. 4	2820	5169	3205	7186	280	14.2
Exp. 5	2993	3505	2264	6701	276	14.3
Mean		5172	3175	7121	292	14.4
SD		1052	597	352	17	1.4
RSD (%)		20.3	18.8	4.9	1.6	10.0
