# Supplementary material for: A new biomarker candidate for spinal muscular atrophy: Identification of a peripheral blood cell population capable of monitoring the level of survival motor neuron protein
Source: PLoS One. 2018 Aug 13;13(8):e0201764. doi: 10.1371/journal.pone.0201764 (PMC6089418; doi:10.1371/journal.pone.0201764)
Supplement: S4 Table — (PDF) [file pone.0201764.s008.pdf]

Supporting Information, Table S4

Table S4 Raw data of median fluorescence intensity (MFI) and spot analysis in CD33^{++} cells (R4)

	Subject ID	Age	Sex	Intracellular ΔMFI	Nuclear ΔMFI	Percentage of Spot^{+} Cells (%)	SMN Spot MFI	Spot^{+} Cells MFI	Mean of Spot count
Control subjects	C1	1y1m	M	6662	4135	20.7	472	6705	0.21
	C2	1y1m	F	7175	4593	28.4	577	7473	0.30
	C3	1y10m	F	9175	5988	41.3	634	8398	0.46
	C4	1y10m	F	8791	5614	43.4	696	8835	0.50
	C5	1y10m	M	6894	4615	15.9	393	6564	0.16
	C6	3y	M	9776	5955	42.8	731	8554	0.50
	C7	5y	F	10252	6217	34.4	641	9236	0.39
	C8	7y	M	9712	5668	28.8	674	8933	0.31
	C9	29y	F	4300	3305	37.7	501	9895	0.52
	C10	31y	F	5642	3310	26.5	488	5757	0.28
	C11	35y	M	5906	3257	25.0	560	6473	0.26
	C12	37y	M	8280	4813	27.9	378	7657	0.34
	C13	38y	F	7023	4688	24.9	540	7156	0.26
	C14	38y	F	7308	4508	33.0	607	8383	0.37
	Mean			7636	4762	30.8	564	7858	0.35
	SD			1753	1023	8.4	108	1216	0.1
SMA subjects	S1	0y2m	F	4083	2463	2.8	317	5011	0.03
	S2	1y2m	M	6713	4657	23.2	435	7719	0.24
	S3	2y2m	M	4914	3300	7.5	276	4719	0.08
	S4	2y10m	M	4381	2509	7.0	330	5169	0.07
	S5	3y9m	F	6820	4538	8.2	339	6700	0.08
	S6	3y10m	F	5821	3635	13.6	445	5950	0.14
	S7	5y	M	6976	4462	12.7	423	7365	0.13
	S8	7y	M	3831	2394	7.6	350	4264	0.08
	S9	7y	F	5774	3655	14.8	376	6236	0.15
	S10	21y	M	4530	2763	5.0	302	5357	0.05
	S11	41y	M	4470	2584	18.1	345	6743	0.20
	S12	2y3m	M	5229	3139	15.8	383	6223	0.16
	S13	3y	F	6734	4457	8.0	374	7040	0.08
	S14	3y2m	M	5087	3504	6.5	343	5969	0.07
	S15	3y5m	M	4852	3032	10.8	377	5476	0.11
	S16	4y	F	5299	3788	11.0	334	6054	0.11
	S17	4y7m	M	5761	3738	10.3	341	6660	0.11
	S18	6y	M	3888	2321	7.0	328	4715	0.07
	S19	8y	F	5612	3729	13.8	346	6181	0.14
	S20	13y	F	2446	1466	11.5	355	5742	0.12
	S21	14y	F	5730	3692	5.3	315	6038	0.05
	S22	7y	F	6041	3966	10.3	348	6099	0.10
	S23	8y	F	5234	3393	16.3	337	5433	0.17
	S24	51y	M	8208	5208	8.7	347	7720	0.09
	S25	60y	F	5914	3638	19.6	427	7175	0.20
	Mean			5374	3441	11.0	356	6070	0.11
	SD			1220	870	5.0	41	931	0.05
	p			*** 3.2E-05	*** 1.3E-04	*** 3.3E-11	*** 2.4E-10	*** 8.8E-06	*** 1.7E-10

(****p* < 1.0E-03)
