# Supplementary material for: A new biomarker candidate for spinal muscular atrophy: Identification of a peripheral blood cell population capable of monitoring the level of survival motor neuron protein
Source: PLoS One. 2018 Aug 13;13(8):e0201764. doi: 10.1371/journal.pone.0201764 (PMC6089418; doi:10.1371/journal.pone.0201764)
Supplement: S5 Table — (PDF) [file pone.0201764.s009.pdf]

Supporting Information, Table S5

Table S5 Summary of the analytical methods for the quantification of the human SMN protein

Analytical method	Sample storage	Sample preparation	Blood volume	SMN protein	Anti-SMN antibody	Molecular interaction	Quantitative capacity (Dynamic Range)	Ref.
Western blot	Cultured fibroblast	Cell Lysis	—	Total SMN	8/SMN	Possible (Not determined)	Semi-quantitative	20
ELISA	PBMC −80°C	Cell Lysis	> 4.0 mL	Total SMN	2B1 SC-15320	Not determined	Quantitative (50 pg–3.2 ng/mL)	18
HTRF	Cultured fibroblast	Cell Lysis	—	Total SMN	Non-disclosure [a)]	Not determined	Quantitative (> 1.0 ng/mL) [a)]	20
ECLIA	Whole blood −80°C	Cell Lysis	3.0 mL	Total SMN	2B1 8/SMN	Not determined	Quantitative (0 pg–50.0 ng/mL) [b)]	8
MSD-ECL	Whole blood −80°C	Cell Lysis	5.0 µL/well	Total SMN	2B1 11708-1-AD	Not determined	Quantitative (0.3 pg–20.0 ng/mL)	22
Imaging flow cytometry	Whole blood RT [c)] 24 h	Hemolyzed blood	< 1.5 mL for $CD33^{++}$ cells	Total SMN Spot analysis	2B1	Possible	Semi-quantitative	Present study

[a)] Product information (Cisbio Bioassays, Codolet, France) [b)] Covering the whole measurement range [c)] RT: Room temperature
