# Supplementary material for: A new biomarker candidate for spinal muscular atrophy: Identification of a peripheral blood cell population capable of monitoring the level of survival motor neuron protein
Source: PLoS One. 2018 Aug 13;13(8):e0201764. doi: 10.1371/journal.pone.0201764 (PMC6089418; doi:10.1371/journal.pone.0201764)
Supplement: S6 Table — (PDF) [file pone.0201764.s010.pdf]

Supporting Information, Table S6

Table S6 The variations in the standard deviation (SD) of the evaluation items with significant differences (***$p < 0.001$)

Evaluation items	Fluorescence intensity (MFI)			Spot Analysis	
	Cell CD33^{++} cells	Nuclei CD33^{++} cells	Nuclei SMN Spot^{+} cells	SMN Spot MFI	Percentage of SMN Spot^{+} Cells
Control (SD)	±1757	±1023	±1216	±108	±8.4
SMA (SD)	±1220	±870	±785	±41	±5.0
Ratio (SMA SD / Control SD)	0.69	0.85	0.77	0.38	0.59
